# Differential Killing of *Salmonella enterica* Serovar Typhi by Antibodies Targeting Vi and Lipopolysaccharide O:9 Antigen

**DOI:** 10.1371/journal.pone.0145945

**Published:** 2016-01-07

**Authors:** Peter J. Hart, Colette M. O’Shaughnessy, Matthew K. Siggins, Saeeda Bobat, Robert A. Kingsley, David A. Goulding, John A. Crump, Hugh Reyburn, Francesca Micoli, Gordon Dougan, Adam F. Cunningham, Calman A. MacLennan

**Affiliations:** 1 School of Immunity and Infection, College of Medicine and Dental Sciences, University of Birmingham, Birmingham, United Kingdom; 2 Wellcome Trust Sanger Institute, Wellcome Trust Genome Campus, Hinxton, Cambridge, United Kingdom; 3 Centre for International Health, University of Otago, Dunedin, New Zealand; 4 Kilimanjaro Christian Medical Centre and Kilimanjaro Christian Medical University College, Tumaini University, Moshi, Tanzania; 5 Division of Infectious Diseases and International Health, Duke University Medical Center, Durham, United States of America; 6 Duke Global Health Institute, Duke University, Durham, United States of America; 7 London School of Hygiene and Tropical Medicine, Keppel Street, London, United Kingdom; 8 Sclavo-Behring Vaccines Institute for Global Health, a GlaxoSmithKline Company, Siena, Italy; 9 Jenner Institute, Nuffield Department of Medicine, University of Oxford, Oxford, United Kingdom; University of Helsinki, FINLAND

## Abstract

*Salmonella enterica* serovar Typhi expresses a capsule of Vi polysaccharide, while most *Salmonella* serovars, including *S*. Enteritidis and *S*. Typhimurium, do not. Both *S*. Typhi and *S*. Enteritidis express the lipopolysaccharide O:9 antigen, yet there is little evidence of cross-protection from anti-O:9 antibodies. Vaccines based on Vi polysaccharide have efficacy against typhoid fever, indicating that antibodies against Vi confer protection. Here we investigate the role of Vi capsule and antibodies against Vi and O:9 in antibody-dependent complement- and phagocyte-mediated killing of *Salmonella*. Using isogenic Vi-expressing and non-Vi-expressing derivatives of *S*. Typhi and *S*. Typhimurium, we show that *S*. Typhi is inherently more sensitive to serum and blood than *S*. Typhimurium. Vi expression confers increased resistance to both complement- and phagocyte-mediated modalities of antibody-dependent killing in human blood. The Vi capsule is associated with reduced C3 and C5b-9 deposition, and decreased overall antibody binding to *S*. Typhi. However, purified human anti-Vi antibodies in the presence of complement are able to kill Vi-expressing *Salmonella*, while killing by anti-O:9 antibodies is inversely related to Vi expression. Human serum depleted of antibodies to antigens other than Vi retains the ability to kill Vi-expressing bacteria. Our findings support a protective role for Vi capsule in preventing complement and phagocyte killing of *Salmonella* that can be overcome by specific anti-Vi antibodies, but only to a limited extent by anti-O:9 antibodies.

## Introduction

*Salmonella enterica* serovar Typhi (*S*. Typhi) is the causative agent of the systemic disease typhoid fever. Humans are the only known natural hosts of *S*. Typhi [1] with an estimated 12 to 22 million cases of typhoid fever per annum resulting in between 129,000 and 217,000 deaths [2, 3].

The majority of clinical *S*. Typhi isolates express a polysaccharide capsule (Vi CPS) known as Vi (Vi^+^ *S*. Typhi) [4] which is associated with virulence [5, 6]. Vi exhibits immunomodulatory activities. For example, Vi expression on *Salmonella* can reduce the inflammatory response in intestinal epithelial cells [7] and throughout systemic infection. Such signatures include reduced levels of growth related oncogene α (GROα) and IL-17, and reduced fluid influx and neutrophil recruitment in the *Salmonella* bovine ileal loop model [8]. Vi expression can thus facilitate a stealth mode of pathogenesis promoting systemic spread and limiting the clinical signatures of gastroenteritis [9]. Vi expression can also reduce the deposition of C3 on the surface of *S*. Typhi and provide protection against non-specific antibody killing [6, 10].

In addition to Vi capsule, *S*. Typhi also expresses the O:9 antigen of lipopolysaccharide, a feature shared with other clinically important *Salmonella* serovars, particularly *S*. Enteritidis. Antibodies to O:9 have bactericidal potential, reducing bactericidal loads post challenge with *S*. Enteritidis, and consequently O:9-based conjugate vaccines are in development to prevent invasive nontyphoidal *Salmonella* disease in Africa [11]. Characterizing the action of anti-O:9 antibodies against *S*. Typhi, and the effect of Vi expression on activity of these antibodies could help understand what potential O:9 conjugate vaccines might have in protection against typhoid fever.

Three licensed vaccine types have been used to prevent typhoid fever [12]. Inactivated whole cell vaccines have some efficacy, but these are associated with high reactogenicity. Ty21a is a live attenuated oral vaccine derived from *S*. Typhi Ty2, that does not express a Vi polysaccharide capsule due to mutations within the Vi locus (*viaB*). While the Ty21a typhoid vaccine lacks Vi capsule, it is unclear whether the protection afforded by this vaccine is mediated by anti-O:9 antibodies.

A third type of vaccine is based on Vi CPS administered intramuscularly. Ty21a and Vi CPS have similar limited cumulative efficacies of approximately 55% at 3 years post-vaccination, despite being based on different antigens and formulations. These vaccines are not recommended for use in children under two years of age [12]. Both Ty21a and Vi CPS vaccines require refrigeration and neither has been implemented in national public health programs. An effective vaccine that can be used in infants remains a public health priority, and new Vi glycoconjugate vaccines are currently in development [12, 13].

This study uses both wild-type *S*. Typhi and pairs of isogenic Vi^+^ and Vi^-^ derivatives of *S*. Typhi and *S*. Typhimurium [14] to investigate the relationship between the Vi capsule and antibody-dependent immunity to *Salmonella*, in particular the role of human anti-Vi and anti-O:9 antibodies.

## Results

### Vi capsule increases resistance of *S*. Typhi and *S*. Typhimurium to antibody-dependent complement-mediated killing by human serum

**Fig 1:**
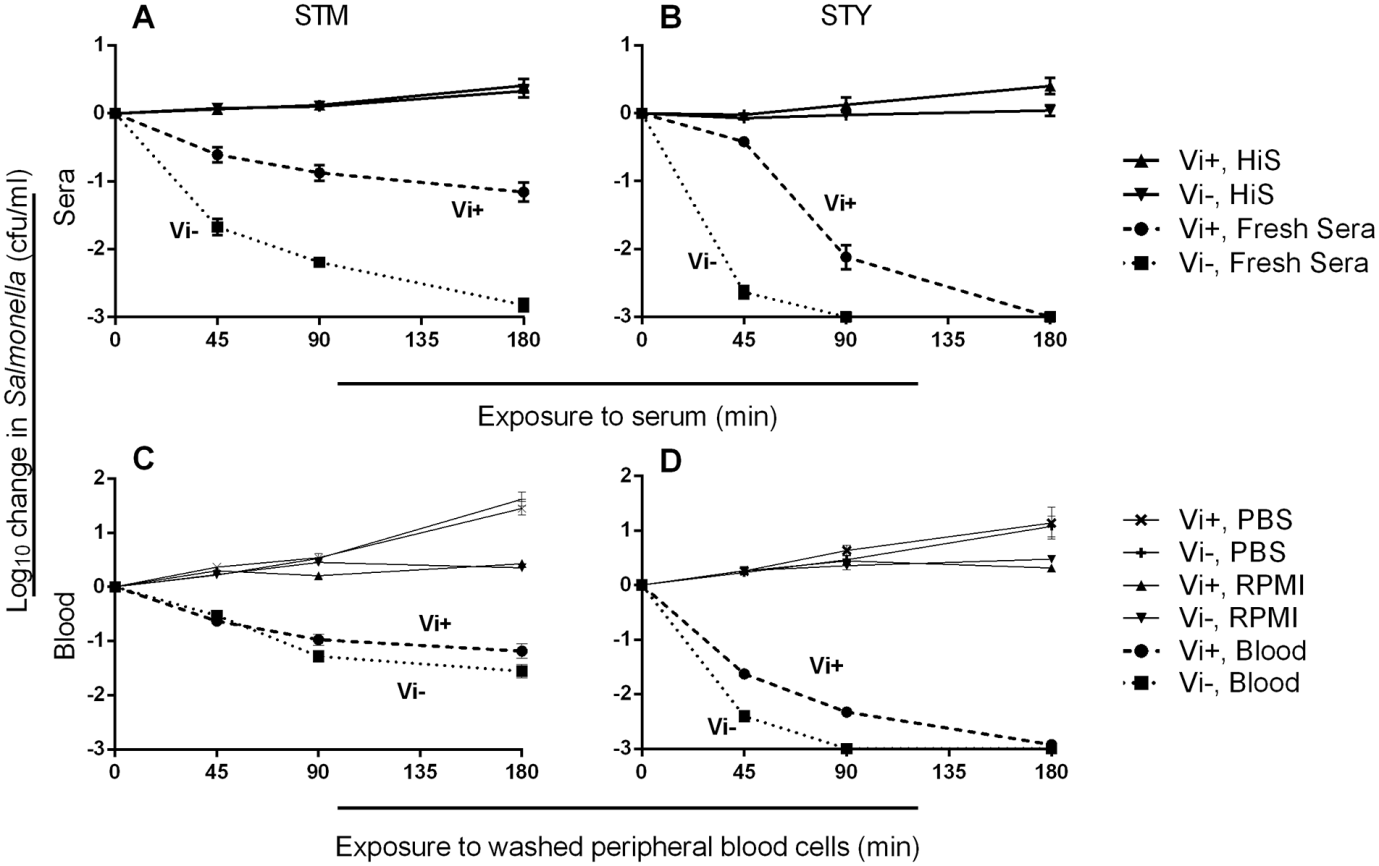
Vi increases serum resistance of *Salmonella* and reduces killing of *Salmonella* by peripheral blood phagocytes. Killing of isogenic Vi-expressing and non-expressing *S*. Typhimurium (A) and *S*. Typhi (B) strains by ten individual adult sera characterized for anti-*Salmonella* antibody content in S1 Fig. For blood cell killing, isogenic Vi-expressing and non-expressing *S*. Typhimurium (C) and *S*. Typhi (D) were opsonized with antibodies from ten adult sera and C6-deficient serum then added to washed peripheral blood cells. Killing of *Salmonella* strains expressing (dashed lines) or not expressing (dotted lines) Vi capsule was recorded at 45, 90 and 180 minutes. C6-deficient serum was used as the source of complement, because it permits opsonization with C3b, but does not result in membrane attack complex formation. Solid lines in A and B are Vi^+^ and Vi^-^ bacteria incubated with heat-inactivated sera (HiS). Solid lines in C and D represent *Salmonella* incubated with PBS and added to blood cells, or opsonized with serum and added to RPMI. Lines represent mean values using sera from ten adults in triplicate experiments. Error bars represent SEM.

We performed bactericidal assays using sera from ten healthy adult donors (S1 Table and S1 Fig) and isogenic Vi^+^ and Vi^-^ isolates of *S*. Typhi and *S*. Typhimurium. Vi expression by both *S*. Typhimurium (Fig 1A) and *S*. Typhi (Fig 1B) enhanced bacterial survival indicating that the protective effect of Vi against complement-mediated killing is not restricted to the serovar Typhi. Vi^+^ C5.507 *S*. Typhimurium was more resistant to killing compared to the isogenic Vi^-^ strain, SGB1 (*p*<0.0001, all time points), and Vi^+^ *S*. Typhi BRD948 was more resistant to killing at 45 and 90 minutes compared with Vi^-^ *S*. Typhi BRD948 (*p*<0.0001, both time points). Both Vi^+^ and Vi^-^ *S*. Typhi BRD948 derivatives underwent a 3 log_10_ kill by 3 hours and were inherently more sensitive to complement-mediated killing than their Vi^+^ and Vi^-^ *S*. Typhimurium counterparts.

### Vi capsule increases the resistance of *S*. Typhi and *S*. Typhimurium to antibody-dependent phagocyte-mediated killing

Blood cell killing assays were performed using the isogenic *Salmonella* isolates opsonized with heat-inactivated serum and fresh C6-deficient serum. C6-deficient serum was used as the source of complement, because it permits opsonization with C3b, but does not result in membrane attack complex formation. Vi^+^ and Vi^-^ *S*. Typhi BRD948 were both more inherently susceptible to phagocyte-mediated killing than Vi^+^ and Vi^-^ *S*. Typhimurium. Vi^+^ *S*. Typhimurium C5.507 was more resistant to blood cell killing than Vi^-^ *S*. Typhimurium SGB1 after 90 and 180 minutes (*p* = 0.042 and 0.047 respectively) (Fig 1C). Vi^+^ *S*. Typhi BRD948 was markedly more resistant to phagocyte killing than Vi^-^ *S*. Typhi BRD948 at 45 and 90 minutes (*p*<0.01) (Fig 1D).

### Vi expression reduces damage to *Salmonella* following exposure to serum

**Fig 2:**
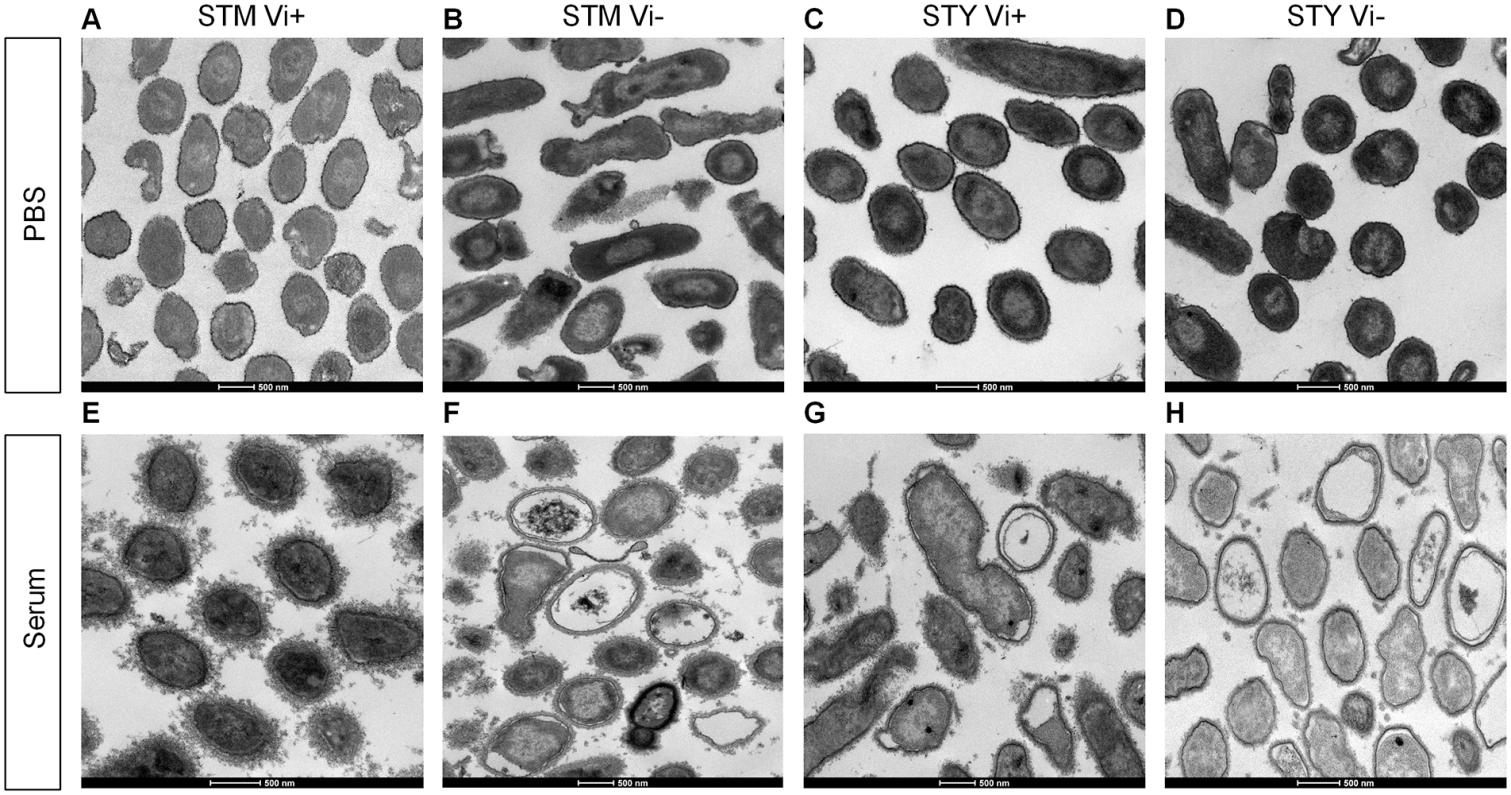
Vi capsule protects against cell damage following serum exposure. Cellular integrity of and damage to *Salmonella* was assessed by transmission electron microscopy (TEM) after exposure to PBS or human sera. TEM images of isogenic (A and E) Vi^+^ *S*. Typhimurium, (B and F) Vi^-^ *S*. Typhimurium, (C and G) Vi^+^ *S*. Typhi and (D and H) Vi^-^ *S*. Typhi after incubation with either PBS (A–D respectively) or with undiluted human serum from Donor 1 (E–H respectively) for ten minutes. Representative images shown.

Damage to Vi-expressing and non-expressing *Salmonella* following exposure to human serum was explored using serum from donor 1, because it contains IgG antibodies to Vi, O:9 and O:4,5, and was visualized by transmission electron microscopy (TEM). For all isolates, exposure to PBS for 10 minutes had no obvious effect on cellular integrity (Fig 2A–2D). Vi^+^ *S*. Typhimurium C5.507 exhibited minimal signs of cellular damage following serum exposure for 10 minutes (Fig 2E), but accumulated a distinct, irregular outer layer on the cell surface, consistent with complement deposition. Both Vi^-^ *S*. Typhimurium C5.507 and Vi^+^ *S*. Typhi BRD948 exhibited similar signs of damage, including enlargement of the periplasmic space with cytoplasmic contraction (Fig 2F and 2G respectively) while Vi^-^ *S*. Typhi cells showed extensive damage (Fig 2H). After scoring multiple images (n = 3), more Vi^-^ *S*. Typhimurium and Vi^-^ *S*. Typhi cells displayed indicators of damage compared to their Vi-expressing counterparts (*p* = 0.008 and 0.004 respectively) (S2 Fig). Hence, the expression of a Vi capsule is associated with reduced damage after short-term exposure to serum, even in the presence of anti-Vi antibodies.

### Vi capsule reduces antibody binding and complement deposition in the absence of Vi-specific IgG

**Fig 3:**
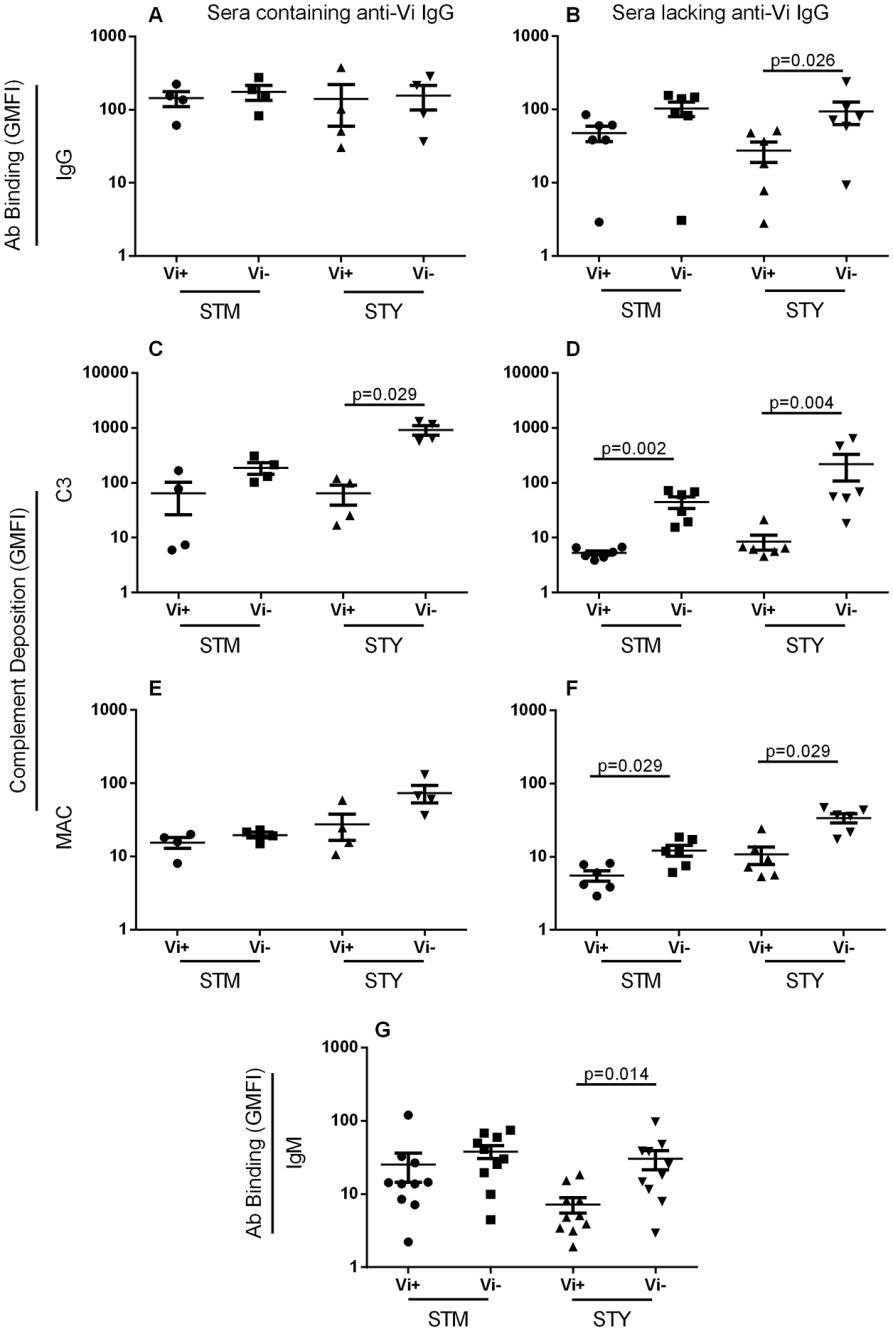
Vi expression decreases antibody and complement binding to *Salmonella* in the absence of specific antibodies. Levels of (A and B) IgG, (C and D) C3 and (E and F) C5b-9 MAC on Vi-expressing and non-expressing *Salmonella* Typhimurium (STM) and *S*. Typhi (STY) after incubation with sera containing anti-Vi IgG (A, C and E) or lacking anti-Vi IgG (B, D and F) were measured by flow cytometry. Levels of IgM binding to Vi^+/-^ *Salmonella* for all ten sera were also assessed (G). All sera contained IgM anti-Vi antibodies. Bars represent mean +/- SEM

We measured total IgG and IgM binding, and C3 and MAC deposition on the isogenic Vi^+^ and Vi^-^ *Salmonella* isolates, following independent incubation with the ten human sera in S1 Table. When incubated with the four sera containing anti-Vi IgG, levels of total IgG binding to both Vi^+^ *S*. Typhi and Vi^+^ *S*. Typhimurium were not significantly different compared with their isogenic Vi^-^ partner strains (Fig 3A). When incubated with the six sera lacking Vi-specific IgG, there was an increase in median total IgG binding to Vi^-^ SGB1 compared with Vi^+^ C5.507 *S*. Typhimurium, although this did not reach statistical significance with the number of samples involved. In the absence of anti-Vi IgG, significantly less IgG bound to Vi^+^ *S*. Typhi BRD948 compared with the isogenic Vi^-^ counterpart (*p* = 0.026) (Fig 3B).

With sera containing anti-Vi IgG, no significant difference in C3 deposition was detected between Vi^+^ *S*. Typhimurium C5.507 and Vi^-^ *S*. Typhimurium SGB1, while significantly lower C3 deposition occurred on Vi^+^ *S*. Typhi compared with its Vi^-^ equivalent (*p* = 0.029) (Fig 3C). In the absence of anti-Vi IgG, expression of Vi was associated with significantly reduced C3 deposition on both Vi^+^ *S*. Typhimurium C5.507 and Vi^+^ *S*. Typhi BRD948 compared with the isogenic Vi^-^ equivalents (*p* = 0.002 and 0.004 respectively) (Fig 3D). No differences in levels of MAC deposition were detected between Vi-expressing and non-expressing *Salmonella* when anti-Vi IgG was present in the sera, although a trend for increased MAC deposition was observed with Vi^-^ *S*. Typhi (Fig 3E). With sera lacking anti-Vi IgG, expression of Vi was associated with significantly reduced MAC deposition on both *S*. Typhimurium C5.507 and *S*. Typhi BRD948 (*p* = 0.026 and 0.009) (Fig 3F). Binding of total IgM from the ten sera was significantly lower for Vi^+^ *S*. Typhi BRD948 compared with the isogenic Vi^-^ counterpart (*p* = 0.014), but not for Vi^+^ *S*. Typhimurium C5.507 compared with the Vi^-^ equivalent (Fig 3G). All ten sera contained IgM anti-Vi antibodies. Incubation with PBS followed by each secondary antibody was performed as a negative control (S3 Fig).

### Depletion of serum anti-Vi antibodies reduces killing of Vi-expressing *Salmonella*

**Fig 4:**
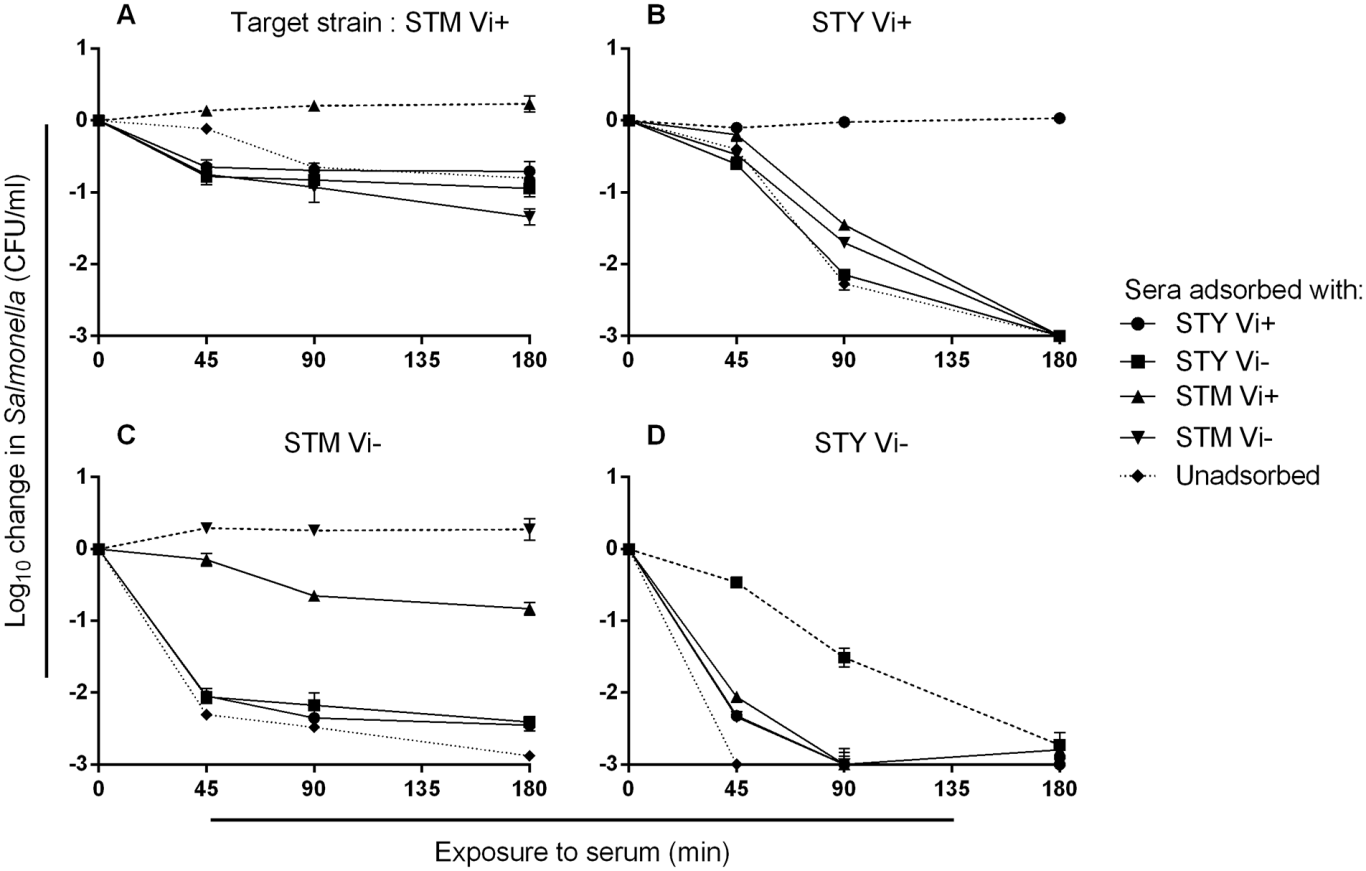
Sera depleted of *Salmonella*-specific antibodies effect variable killing of different *Salmonella* strains. Killing of Vi^+^ *S*. Typhimurium (STM) (A) and *S*. Typhi (STY) (B) and Vi^-^ *S*. Typhimurium (C) and *S*. Typhi (D) by donor 1 serum adsorbed of antibodies with Vi^+^ and Vi^-^ *S*. Typhimurium and *S*. Typhi or unadsorbed serum. Dashed lines represent killing of *Salmonella* by sera adsorbed using the homologous *Salmonella* strain. Dotted lines represent killing by unadsorbed serum. Experiments were performed in triplicate and error bars represent SEM. Initial concentration of bacteria in each assay was 10^6^ CFU/ml.

To investigate the functional role of anti-Vi antibodies in killing of *Salmonella*, donor 1 serum was used as it contains IgG antibodies to Vi, O:9 and O:4,5. This was adsorbed by incubation with each of the two pairs of isogenic Vi^+^ and Vi^-^ *S*. Typhimurium and *S*. Typhi isolates, leading to selective depletion of antibodies to Vi, O:9 and O:4,5 (S4 Fig). With the exception of Vi^-^ *S*. Typhi, which is sensitive to killing by complement in the absence of specific antibodies, adsorbed serum was unable to kill the isolate used for adsorption (homologous isolate) (Fig 4). Adsorbed sera were able to kill all other isolates (heterologous isolates), whether heterologous by serovar or for Vi expression. Hence, sera adsorbed with Vi^-^ *Salmonella* could still kill the isogenic Vi^+^ *Salmonella* strain of the same serovar (*p* = 0.005 and <0.0001 for *S*. Typhimurium and *S*. Typhi respectively) (Fig 4A and 4B). This is consistent with complement-mediated killing secondary to binding of anti-Vi antibodies. Similarly, sera adsorbed with the Vi^+^ *Salmonella* could kill the Vi^+^ *Salmonella* derivative of the heterologous serovar (*p* = 0.018 and 0.0003 for *S*. Typhimurium and *S*. Typhi respectively). This is consistent with killing of Vi^+^ *Salmonella* by non-Vi antibodies such as anti-O:9 and anti-O:4,5 and suggests that the Vi capsule does not fully prevent access of non-Vi antibodies to their targets.

### Altered osmolarity reduces Vi expression, enhancing anti-O:9 antibody binding to wild-type *S*. Typhi

**Fig 5:**
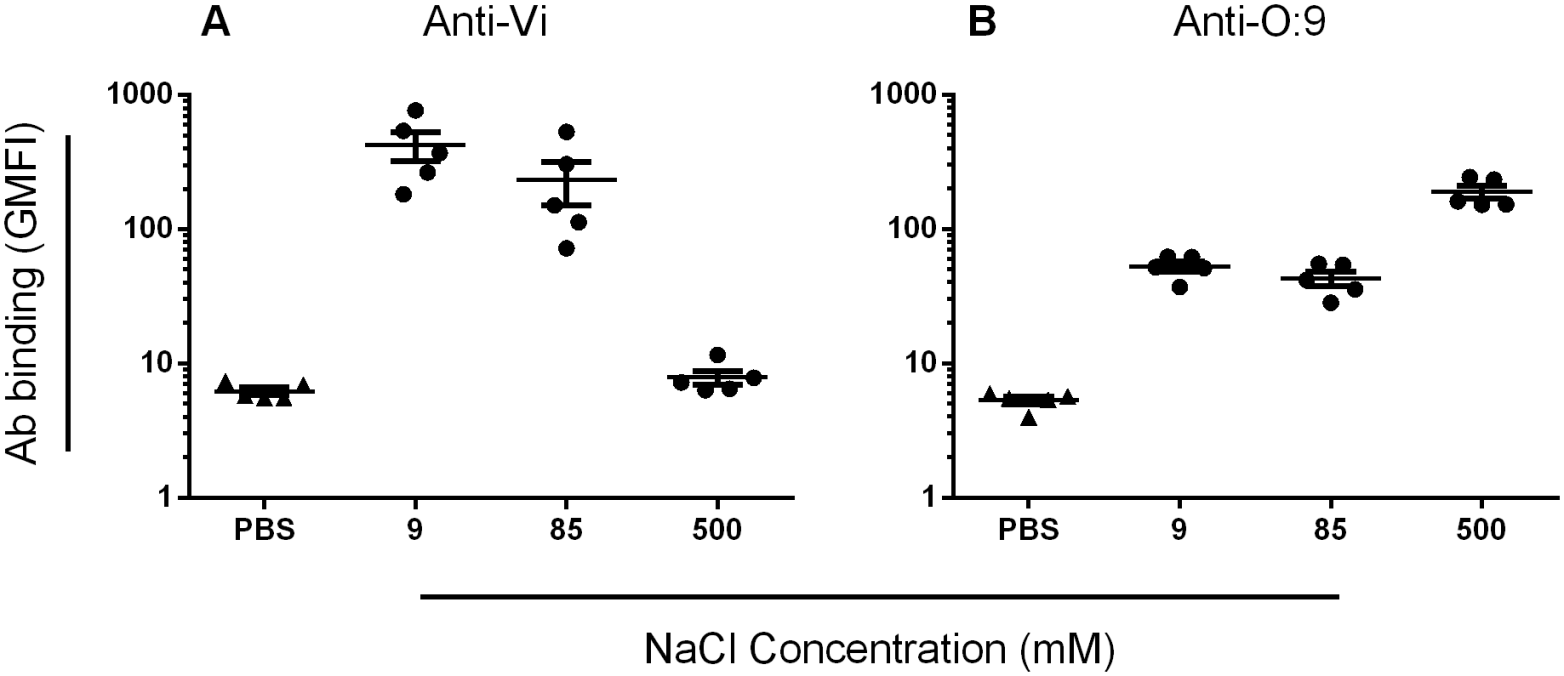
Media osmolarity affects the binding of human anti-Vi and anti-O:9 antibodies to wild-type *S*. Typhi. Five wild-type *S*. Typhi isolates, one from each geographical site to ensure the greatest geographical variation among strains studied, were cultured in LB broth containing 9, 85 or 500mM NaCl and incubated with either purified human anti-Vi (A) or anti-O:9 (B) antibodies or PBS. Levels of antibody binding to *S*. Typhi were assessed by flow cytometry. Values are mean of three separate experiments. Bars represent mean +/- SEM.

Vi expression on *Salmonella* is influenced by osmolarity, surface expression being down-regulated as osmolarity increases [14, 15]. We affinity purified anti-Vi and anti-O:9 antibodies from a pool of sera from three healthy adults who had been vaccinated with Vi CPS (S5 Fig) and used these to examine the effect of altered Vi expression on antibody binding. Five different wild-type *S*. Typhi isolates were cultured at 9, 85 and 500 mM NaCl in order to modulate expression of the Vi capsule. Anti-Vi antibody binding to *S*. Typhi was highest when *Salmonella* was cultured at 9mM NaCl and minimal when cultured at 500 mM NaCl (Fig 5A), consistent with increased Vi expression at low osmolarity and the converse at high osmolarity. Anti-O:9 antibody binding to *S*. Typhi was highest at 500 mM NaCl, where Vi expression is minimal, most likely due to enhanced access to target antigen, unimpeded by Vi capsule. Anti-O:9 binding in 85 and 9 mM NaCl, where Vi expression is increased, was significantly lower (*p* = 0.008 for both), but still detectable (p<0.007 for both compared with negative control) (Fig 5B) Thus, there was a negative correlation between anti-Vi and anti-O:9 antibody binding to *S*. Typhi isolates cultured at 9, 85 and 500mM NaCl (S6 Fig) (r = -0.62, *p* = 0.016). These findings are consistent with Vi capsule limiting access of anti-O:9 antibodies.

### Altered osmolarity and Vi expression modulates killing by anti-Vi and anti-O:9 antibodies

**Fig 6:**
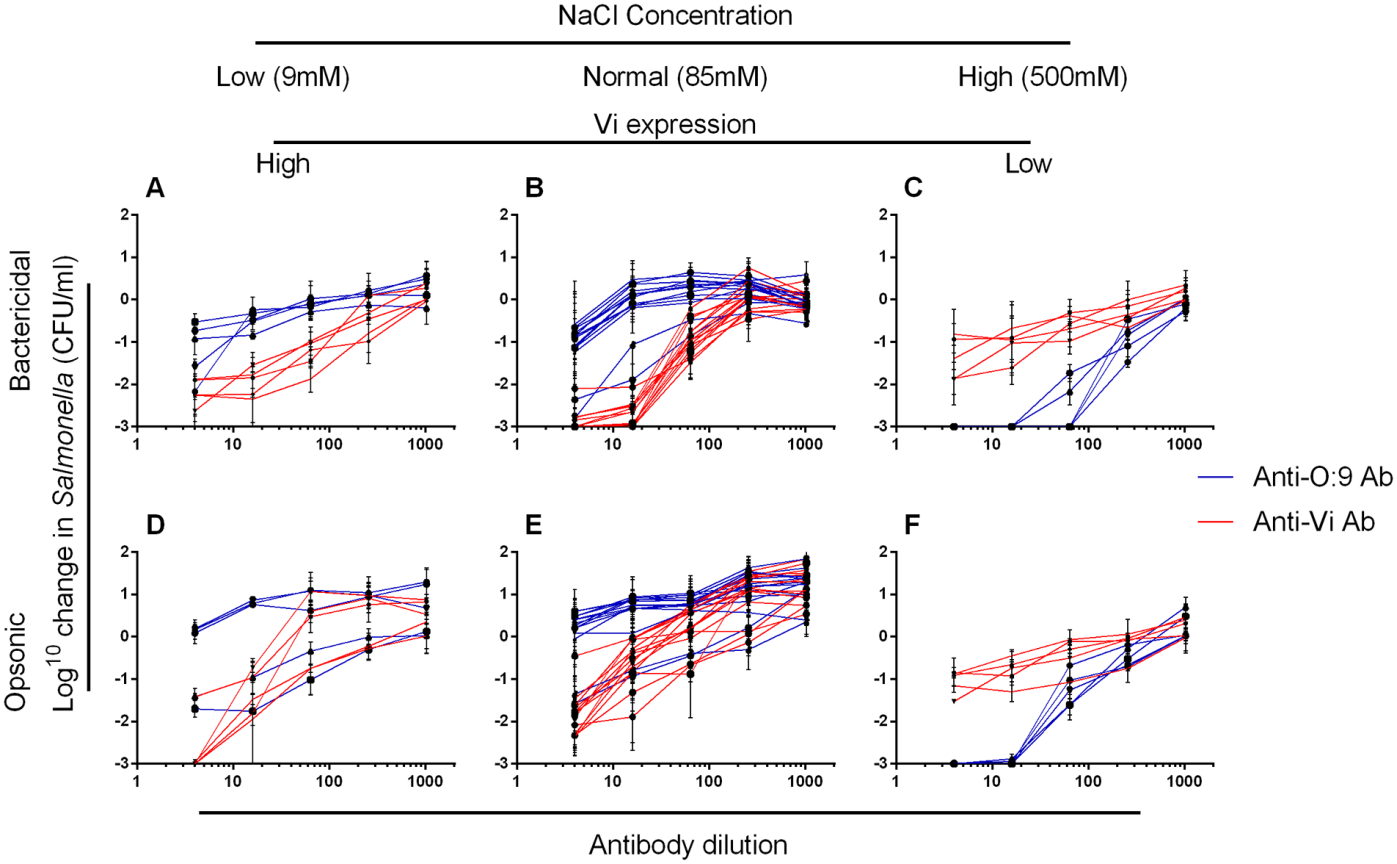
Bactericidal and opsonic activity of anti-Vi and anti-O:9 antibodies against *S*. Typhi depends on medium osmolarity. The bactericidal (A–C) and opsonic (D–F) activity of purified human anti-Vi (red lines) and anti-O:9 (blue lines) antibodies supplemented with human complement was tested against wild-type *S*. Typhi. *S*. Typhi isolates were cultured in LB Broth containing 9 (high Vi expression, A and D), 85 (B and E) or 500mM (low Vi expression, C and F) NaCl. Isolates tested at 9mM or 500mM NaCl concentrations were the same and included in the 14 isolates tested at 85mM NaCl. They were selected from each geographical site to ensure the greatest geographical variation among strains studied. Each line represents an individual *S*. Typhi isolate. Data represent mean of three separate experiments +/- SEM.

We assessed the bactericidal and opsonic potential of purified human anti-Vi and anti-O:9 antibodies against 14 wild-type *S*. Typhi isolates cultured in ordinary LB broth (85mM NaCl), and five (one from each geographical site to ensure the greatest geographical variation among strains studied, and the same five as used in Fig 5) cultured in LB broth at 9 and 500 mM NaCl (Fig 6). Anti-Vi and anti-O:9 antibodies were bactericidal against *S*. Typhi. Anti-Vi antibodies killed *S*. Typhi with normal (Fig 6B) or enhanced (Fig 6A) Vi expression more effectively than anti-O:9 antibodies. With minimal Vi expression, the effect was reversed, with anti-O:9 antibodies effecting a 3 log_10_ kill of all five *S*. Typhi isolates and reduced bactericidal activity of anti-Vi antibodies (Fig 6C). Anti-O:9 antibodies poorly facilitated opsonophagocytic killing of *S*. Typhi expressing normal or elevated amounts of Vi, being unable to kill all but two *S*. Typhi strains. In contrast, *S*. Typhi with normal or elevated Vi and opsonized with anti-Vi antibodies were effectively killed by blood phagocytes (Fig 6D and 6E). The opsonic activity of anti-O:9 antibodies against *S*. Typhi was greater than that of anti-Vi antibodies when *Salmonella* had reduced Vi expression (Fig 6F).

### MAC deposition in the presence of anti-Vi antibodies, but not anti-O:9 antibodies, correlates with killing of *S*. Typhi in the serum bactericidal assay

**Fig 7:**
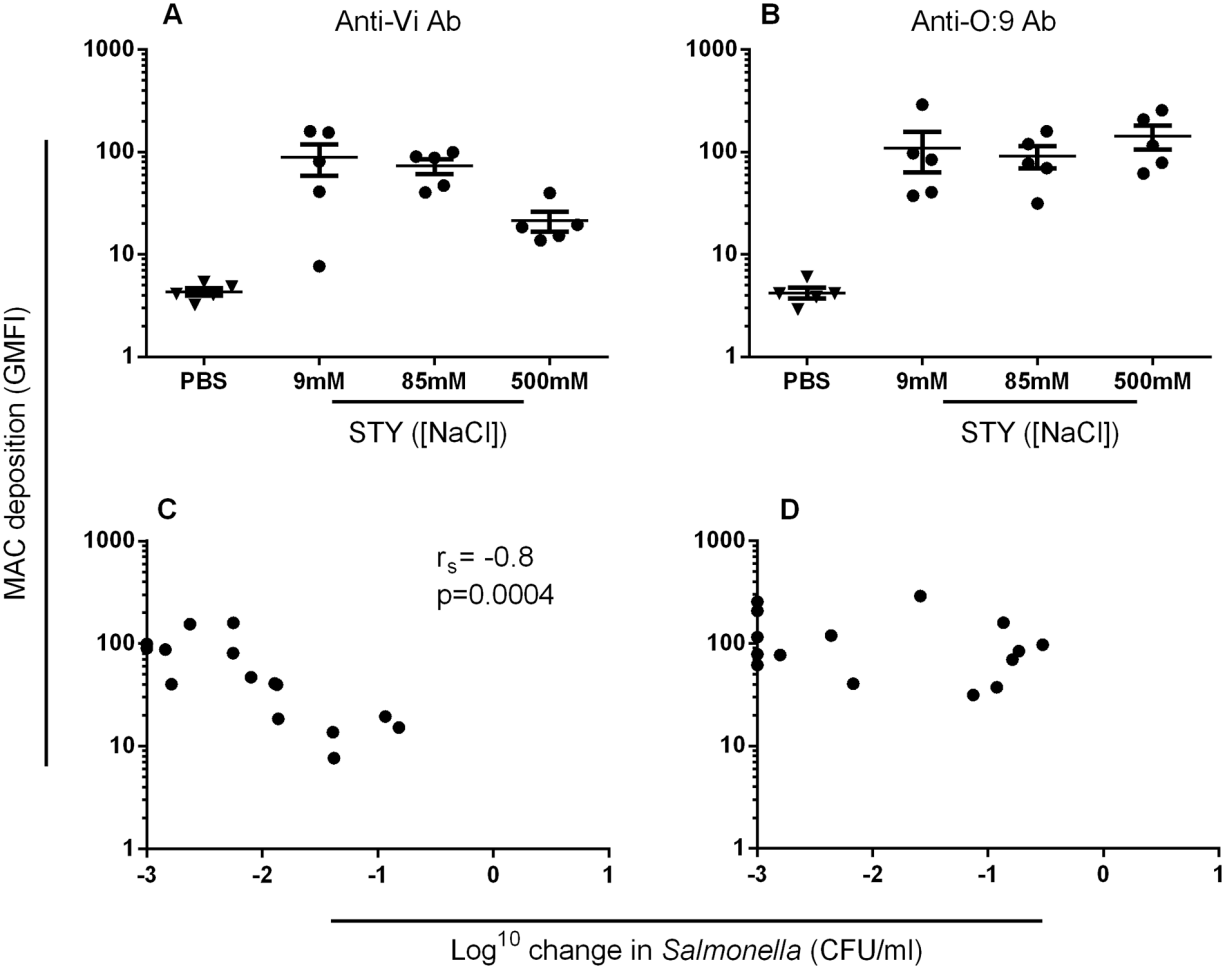
MAC deposition in the presence of anti-Vi, but not anti-O:9 antibodies, is associated with bactericidal activity. Levels of C5b-9 MAC deposition on the surface of five wild-type *S*. Typhi isolates cultured at 9, 85 and 500mM NaCl in the presence of purified human anti-Vi antibodies (A) or anti-O:9 antibodies (B) were measured by flow cytometry. For each of the five isolates at each concentration of sodium chloride, killing in the serum bactericidal assay was plotted against the level of MAC deposition for anti-Vi antibodies (C) or anti-O:9 antibodies (D). Data are mean of three separate experiments. Bars represent mean +/- SEM.

We assessed MAC deposition in the presence of purified anti-Vi and anti-O:9 antibodies on *S*. Typhi expressing different amounts of Vi through changes in osmolarity. For anti-Vi antibodies, MAC deposition was highest when conditions favored Vi expression on *Salmonella*, and decreased as Vi expression was reduced (Fig 7A). Somewhat surprisingly, there were similar levels of MAC deposition with anti-O:9 antibodies irrespective of the level of expression of Vi (Fig 7B). In the presence of anti-Vi antibodies, MAC deposition correlated with killing (Spearman’s r = -0.8, p = 0.0004) (Fig 7C). However, there was no correlation between levels of MAC deposition with anti-O:9 antibodies and killing of *S*. Typhi (Fig 7D). This suggests that MAC deposition secondary to binding of O:9 antibodies is not effective at killing in the presence of high levels of Vi antigen.

## Discussion

We have examined the relationship between Vi polysaccharide expression in *Salmonella* and sensitivity to killing by antibody, complement and blood phagocytes from healthy adults. We demonstrate that antibodies to Vi can be bactericidal and opsonic and kill Vi-expressing *S*. Typhi and *S*. Typhimurium. The level of killing correlated with Vi expression.

Our findings indicate an inherent difference in resistance to complement-mediated killing between Typhi and Typhimurium serovars. Vi^-^ *S*. Typhi was considerably more sensitive to complement-mediated killing than the equivalent *S*. Typhimurium and can be killed by complement alone in the absence of specific antibodies. This may explain why the majority of *S*. Typhi that cause disease express a Vi capsule. Our observation that Vi expression by *Salmonella* is associated with an increase in serum resistance is in agreement with previous findings [6, 10], but contrasts with work by *Bravo et al* [16]. This may be a consequence of differing methods with *Bravo et al* limiting their serum bactericidal assay to 20 minutes with a maximum serum concentration of 20%.

*S*. Typhimurium was compared with *S*. Typhi, because *S*. Typhimurium is the commonest serovar responsible for invasive nontyphoidal *Salmonella* disease, while *S*. Typhi is the most common serovar responsible for enteric fever [12, 17]. Therefore, these are the two *Salmonella* serovars most in need of a vaccine and so the serovars for which it is most important to understand the basis of protective immunity in man. Similar findings to those obtained with *S*. Typhimurium would be expected with *S*. Enteritidis, since both serovars show a comparable level of resistance to antibody-mediated killing in serum [18].

As previously shown with O-antigen-expressing invasive clinical *S*. Typhimurium isolates from Africa [19], Vi^-^ *S*. Typhimurium is not susceptible to killing by complement alone and requires more complement than other *Salmonella* serovars to be killed [20]. Killing of Vi^-^ *S*. Typhimurium by undiluted, control adult human serum is more gradual than for Vi^-^ *S*. Typhi. Unlike the Vi^-^ equivalent, Vi^+^ *S*. Typhi BRD948 demonstrated similar resistance to killing by healthy human adult serum as Vi^-^ *S*. Typhimurium SGB1 with both these strains incurring similar levels of structural damage following exposure to serum (Fig 2). If, as we have previously suggested [21], such a level of serum resistance is optimal for *Salmonella* to successfully survive as a pathogen in man, this could explain in part why the inherently more complement-susceptible *S*. Typhi have a Vi capsule, while *S*. Typhimurium do not.

We found a significant reduction in MAC deposition on the Vi^+^ *S*. Typhimurium and Typhi compared to their respective isogenic Vi^-^ counterparts when exposed to sera lacking anti-Vi IgG. A reduction in C3 deposition, under the same conditions, is in agreement with previous work [6, 10]. Total IgG and IgM binding to Vi-expressing *S*. Typhi, was significantly lower than to Vi^-^ *S*. Typhi, particularly when sera lacked anti-Vi IgG. Differences in the level of Vi expression between Vi^+^ *S*. Typhi and *S*. Typhimurium, with relatively lower Vi expression by the *S*. Typhimurium strain, may partly explain why Vi expression was not associated with significant reductions in IgG and IgM binding to *S*. Typhimurium. However, inactivation of *fepE* is common in *S*. Typhi and results in the loss of very-long O-antigen chains [22], reducing the opportunity for binding of anti-O-antigen antibodies, particularly in the presence of a Vi capsule.

These findings are consistent with the concept that Vi expression impedes complement-mediated killing, either by 1. restricting binding of antibodies to the *Salmonella* surface, 2. restricting binding of antibodies to O-antigen, 3. directly reducing the deposition of complement factors, 4. diverting complement deposition to sites where it is not bactericidal or 5. a combination of these mechanisms. A reduction in complement deposition resulting from the modification of surface structures has been shown to result in increased resistance to killing in other bacteria including *E*. *coli* K1 [23] and *N*. *meningitidis* [24].

Purifying human anti-Vi and anti-O:9 antibodies allowed us to explore the role of each type of antibody in killing of *S*. Typhi in greater detail. By altering the medium osmolarity, we were able to vary expression of Vi and investigate how this affected antibody-mediated killing. Human anti-Vi antibodies effected bactericidal and opsonic activity against wild-type *S*. Typhi under conditions of Vi capsule expression. The bactericidal, but not the opsonophagocytic killing activity of anti-Vi antibodies was slightly reduced at 9mM NaCl, where Vi expression is greatest, compared to 85mM NaCl. This is consistent with the Vi capsule having intrinsic properties which augment resistance to killing by the membrane attack complex, a structure which is not involved in opsonophagocytic killing, despite the presence of capsule specific antibodies. Under the same conditions, anti-O:9 antibodies exerted some bactericidal activity against *S*. Typhi, but were poorly able to facilitate opsonophagocytic killing. With loss of Vi capsule expression at 500mM NaCl, binding, bactericidal and opsonic activity of anti-O:9 antibodies was greatly enhanced.

*S*. Enteritidis is one of the most prevalent serovars of NTS globally [25], thus circulating anti-O:9 antibodies are likely to be present in a large proportion of the population. All ten serum donors had detectable anti-O:9 IgG and IgM. However, it has not been known whether the presence of anti-O:9 antibodies confers protection against *S*. Typhi. Our findings suggest that, while anti-O:9 antibodies may effect some complement-mediated bactericidal activity against *S*. Typhi, they are unlikely to contribute to phagocyte-mediated killing in the presence of Vi capsule. Since Vi^LOW^ *S*. Typhi are highly susceptible to both bactericidal and opsonic killing by anti-O:9 antibodies, it appears that Vi mitigates the action of anti-O:9 antibodies, thereby providing another rationale for why the majority of clinical *S*. Typhi isolates express a Vi capsule [4].

Anti-O:9 antibodies were still able to bind to *S*. Typhi under conditions of Vi expression, resulting in MAC deposition. However, while MAC deposition in the presence of anti-Vi antibodies correlated with killing, this was not the case with MAC deposition secondary to anti-O:9 binding. This suggests that Vi capsule does not fully obstruct access to O-antigen allowing both anti-O:9 antibodies to bind and MAC deposition to occur, but without killing. Such differential killing by MAC suggests that the location of MAC deposition on the bacterium is a key determinant for whether *S*. Typhi are killed.

An important implication of these findings relates to the variable expression of Vi [26] and the frequency of anti-O:9 antibodies in humans. Antibodies to nontyphoidal *Salmonella* serovars, such as O:9 expressed by *S*. Enteritidis, are acquired rapidly in the first years of life in regions such as sub-Saharan Africa [19, 27] and are likely present in most adults in all populations later in life. Since Vi antigen is expressed under conditions of low osmolarity, such as would be expected within the bloodstream, it can help protect against killing by these widely prevalent anti-O:9 antibodies aiding the establishment of infection with *S*. Typhi. Similarly, it has previously been shown that the Vi capsule, when expressed by *S*. Typhimurium, reduces, but does not fully remove, protection mediated by antibodies to *Salmonella* porins [28].

In conclusion, these results support the concept that expression of the Vi capsule increases resistance to O:9 and other non-Vi antibodies, complement killing by MAC and opsonization for killing by phagocytes. Modulation of Vi expression has the potential to vary sensitivity during the infection cycle to O:9-specific antibodies induced through exposure to ubiquitous *S*. Enteritidis strains. This may facilitate the establishment of initial systemic infection by *S*. Typhi and subsequent chronic carrier state. The study suggests the existence of a dichotomy whereby Vi expression prolongs *S*. Typhi survival in a naive host through enhanced complement resistance, but in the presence of anti-Vi antibodies, protection is overcome. These findings inform the development of vaccines against typhoid fever and other invasive forms of *Salmonella* disease.

## Materials and Methods

### Bacteria and culture conditions

*S*. Typhimurium were the Vi-negative isolate SGB1 and the Vi-positive isolate C5.507, which harbors the *S*. Typhi *viaB* locus and expresses the Vi polysaccharide capsule [14]. The Vi-positive *S*. Typhi *aro* mutant BRD948 (*S*. Typhi Ty2 *aroC*^-^ *aroD*^-^ *htrA*^-^) and an otherwise isogenic Vi-negative *tviB* mutant derivative were used throughout. Other wild-type *S*. Typhi isolates were from Central Africa, Nepal and Indonesia, and Moshi and Muheza in Tanzania. Bacteria were cultured on LB agar plates or in LB broth and, in the case of *aro* mutants, LB broth and agar were supplemented with tyrosine, tryptophan and phenylalanine at a final concentration of 40 μg/L and para-aminobenzoic acid (PABA) and di-hydroxybenzoic acid at a final concentration of 10 μg/L, as previously reported [29]. For growth at low osmolarity (9mM NaCl), low salt LB broth and agar was used (Sigma). Sodium chloride was added to normal LB Broth and agar to achieve 500mM NaCl.

Vi expression in Vi^+^ strains and its absence in Vi^-^ strains was confirmed by slide agglutination and flow cytometry (S7 Fig) Vi^+^ *S*. Typhi (BRD948) and Vi^+^ *S*. Typhimurium (C5.507) both expressed Vi, with expression lower in *S*. Typhimurium compared with *S*. Typhi. This is consistent with previously reported differential Vi expression levels for these strains [14]. Neither the Vi^-^ *S*. Typhi (BRD948 *tviB*^-^) nor Vi^-^ *S*. Typhimurium strains (SGB1) expressed Vi.

### Serum preparation

Blood was collected from ten healthy adults of mixed age, race and typhoid vaccination history living in the UK (S1 Table) and left to clot at room temperature for 5 h. Three donors had previously received Vi CPS vaccine more than 3 years prior to serum collection. None had a history of suspected or confirmed *Salmonella* disease. Serum was then separated by centrifugation and aliquots stored at -80°C. C6-deficient serum was from an individual with congenital C6 deficiency. When required, heat inactivation of serum was performed at 56°C for 2 hours prior to use. Ethical approval for the use of blood and serum samples in this study was granted by Life and Health Sciences Ethical Review Committee of the University of Birmingham. Informed written consent was obtained from all participants. Anti-*Salmonella* IgG, IgA and IgM antibody profiles against Vi and the O:4,5 (present on *S*. Typhimurium) and O:9 (present on *S*. Enteritidis and *S*. Typhi) LPS-associated antigens were determined in the ten sera by ELISA (S1 Fig). IgG to O:4,5 and O:9 LPS was present in 9/10 sera, while anti-Vi IgG was present in 4/10 sera. IgM antibodies against all three antigens were detected in eight of the ten sera and IgM anti-Vi antibodies in all sera.

### Production of antibody-depleted human sera

*Salmonella*, at a concentration of 1 x 10^11^ CFU/ml, and fresh undiluted human sera were cooled to 4°C. One part bacterial culture was added to nine parts human serum and the mixture incubated on a rocker plate (20 rpm) at 4°C for 1 h. The sera were then centrifuged at 3,300 x g at 4°C for 5 minutes and aspirated into a new tube and the cycle repeated for three incubations. Following the final adsorption, sera were filter-sterilized using a 0.2 μm filter and frozen at -80°C until required.

### Anti-*Salmonella* antibody binding and complement deposition assay

Antibody binding to *Salmonella* was performed as previously described [19]. Briefly, 5 μl of *Salmonella* in PBS/1% formaldehyde was added to 45μl 10% human serum or 45 μl 10% purified human antibodies in PBS (where 100% purified antibodies is equivalent to the concentration of antibodies in the corresponding whole serum) for antibody deposition assays. For complement deposition, *Salmonella* were added to 45μl undiluted human serum or to 45 μl of an antibody/complement mixture (25% antibody, 75% complement) (final bacterial concentration 1 x 10^9^ CFU/ml). Antibody binding was detected using FITC-conjugated anti IgG, IgA or IgM (Sigma) and complement binding detected using FITC-conjugated anti-C3 IgG or mouse anti-C5-9 IgG followed by FITC-conjugated rabbit anti-mouse IgG (DAKO). Samples were analyzed by flow cytometry using a FACSCalibur instrument (BD Biosciences) and Cell Quest Pro software (BD Biosciences). Labeled *Salmonella* were identified based on their forward and side scatter and the geometric mean fluorescence intensity (GMFI) in the FL1 channel recorded as the relative antibody or complement binding level.

### Characterization of Vi expression

*Salmonella* were grown to an OD_600_ of 0.5 in LB broth. Bacteria were washed twice and resuspended in PBS. Mouse anti-Vi antiserum from the Sclavo Behring Vaccines Institute of Global Health (SBVGH, formerly Novartis Vaccines Institute of Global Health) was added to the *Salmonella* at a final concentration of 1:800. Samples were incubated on ice for 1 hour followed by washing with PBS. Allophycocyanin-conjugated rabbit anti-mouse IgG (Sigma) was added at a final concentration of 1:800 and samples incubated for 1 h, washed, and resuspended in PBS/1% formaldehyde. Samples were analyzed by flow cytometry using a FACSCanto instrument (BD Biosciences) and FlowJo software (Tree Star, Inc). *Salmonella* were identified based on forward and side scatter characteristics and the level of Vi expression determined as the GMFI in the FL4 channel.

### Transmission Electron Microscopy (TEM)

Log phase *Salmonella* at a final concentration of 1 x 10^8^ CFU/ml were exposed to fresh human serum (Donor 1, S1 Table) or PBS for 10 minutes at 37°C. Following washing with PBS samples, the bacteria were fixed for 20 minutes with a 1:1 ratio of 5% glutaraldehyde (GA), 1.4% ruthenium hexamine trichloride (RHT) in 0.1M sodium cacodylate buffer (pH 7.42) and 100mM L-Lysine monohydrochloride in PBS. A further fix of 40 minutes using 2.5% GA and 0.7% RHT in sodium cacodylate buffer was applied. Following this, samples were washed in cacodylate buffer and fixed with 1% Osmium tetroxide (Sigma), 0.05M Potassium ferrocyanide and 0.6% RHT in 0.1M sodium cacodylate buffer for 30 minutes at RT. Cells were resuspended in 2% glutaraldehyde (TAAB Laboratories) and 4% paraformaldehyde (Sigma) in PBS for 1 hour at RT, centrifuged at 10,000 rpm for 2 minutes and the supernatant replaced with 0.1M sodium cacodylate and post fixed in 1% osmium tetroxide (TAAB Laboratories) in the same buffer at RT for 1 h. Cells were then dehydrated in an ethanol series, with 1% uranyl acetate added at the 30% stage, followed by propylene oxide and embedded in resin (TAAB Laboratories) prior to polymerization at 60°C for 48 h. Sections were cut on a Leica Ultracut UC6 ultramicrotome at 70nm using a diamond knife, contrasted with uranyl acetate and lead citrate and examined on an FEI Spirit Biotwin 120kV transmission electron microscope and imaged with a Tietz F415 CCD camera.

### Serum bactericidal assays with human sera

Killing of *Salmonella* with fresh, human serum was assessed as previously described [19]. 5 μl *Salmonella* in log growth at a concentration of 1 x 10^7^ CFU/ml was added to 45 μl human serum (final concentration 1 x 10^6^ CFU/ml) and incubated at 37°C for 180 minutes on a rocker plate at 20 rpm. At 45, 90 and 180 minutes, samples were taken and the concentration of viable *Salmonella* was determined by serial dilution and plating on LB agar. Agar plates were incubated overnight at 37°C and colonies counted the following day.

### Blood cell killing assays

Killing of *Salmonella* by peripheral blood cells was based on the assay previously described [30]. 1 x 10^7^ CFU/ml *Salmonella* in log growth were opsonized at a 1:10 ratio with either 50% human serum, which had been heat-inactivated for 2 hours at 56°C, and 50% fresh C6-deficient serum or PBS at RT for 20 minutes. Such C6-deficient serum can act as source of opsonizing complement, but cannot promote C5b-9 membrane attack complex (MAC) formation. Opsonized *Salmonella* were added to blood from healthy adult donors, which had been washed twice with RPMI to remove endogenous antibody and complement, or to RPMI. *Salmonella* were added at a 1:10 ratio to give a final concentration of 1x10^5^ CFU/ml and incubated at 37°C for 180 minutes. Samples were taken at 45, 90 and 180 minutes and the concentration of viable bacteria assessed by serial dilution.

### Serum bactericidal, and blood cell killing assays using purified human antibodies

Serum bactericidal assays and blood cell killing assays using purified human antibodies against the Vi and O:9 antigens of *Salmonella* were conducted as described above. Instead of serum, 11.25 μl anti-Vi and anti-O:9 antibodies (25% final volume) corresponding to 750 and 4268 IgG EU per ml respectively were combined, in a 1:4 dilution series, with 33.75 μl *Salmonella*-antibody-depleted human serum (75% final volume).

### Purification of human antibodies

Affinity purification of human antibodies was performed as previously described [31]. In brief, sera from three healthy adults who had received the Vi CPS were pooled and passed through affinity chromatography columns (HiTrap) containing either 2.5 mg Vi PS or 1 mg O:9 antigen. Both purified polysaccharides were activated with adipic acid dihydrazide and linked to N-hydroxysuccinamide-sepharose resins. Antibodies were sequentially eluted using 0.1 M glycine pH 2.4 and 4M MgCl_2_ pH 7. Eluates were pooled and dialyzed overnight against PBS. Purified antibodies were concentrated using Vivaspin columns (GE Healthcare) with a 10,000 MW cut-off.

### ELISA for anti-*Salmonella* LPS O-antigen and anti-Vi antibodies

96-well flat-bottom plates (NUNC Maxisorp) were incubated overnight at 4°C with 100 μl per well of 5 μg/ml TLR-grade smooth O:4,5 LPS or O:9 LPS (Alexis Biochemicals),1 μg/ml Vi polysaccharide from SBVGH [32] or 1μg/ml outer membrane proteins (OMPs) produced from *S*. Typhimurium SL3261. Plates were washed three times with PBS/0.05% Tween 20 and incubated with 200 μl blocking buffer (PBS/1% BSA) per well at 37°C for 1 h. Plates were washed again and incubated with a threefold dilution series of each sera in dilution buffer (PBS/1% BSA/0.05% Tween 20) for 1 hour at 37°C. Plates were washed again and 100 μl of goat anti-human alkaline phosphatase-labeled IgG, A or M (Southern Biotech) diluted 1:2000 in PBS was added to each well and plates incubated for one hour at 37°C. Finally plates were washed and 100 μl of substrate (Sigma N2770 p-Nitrophenyl phosphate) added to each well. Plates were left to develop at 37°C for 1 h. The reciprocal of the dilution of sera giving an OD_405_ of 1 was determined. This value was plotted as the anti-*Salmonella* LPS (O:4,5 or O:9) or anti-Vi IgG, A or M, concentration.

### Statistical Analysis

Data from experiments using different manipulations of *Salmonella* killing were compared using Student’s t test. Comparisons of antibody binding and complement deposition on *Salmonella* were performed using the Mann-Whitney U test.

### Ethics statement

Ethical approval for the use of blood and serum samples in this study was granted by Life and Health Sciences Ethical Review Committee of the University of Birmingham. Informed written consent was obtained from all participants.

## Supporting Information

S1 Fig Anti-*Salmonella* antibody levels in sera from ten healthy adults. Levels of IgG (A), IgA (B) and IgM (C) against the *Salmonella* antigens LPS O:4,5, LPS O:9 and Vi polysaccharide as measured by ELISA. Lines represent median and interquartile range.
(TIF)

S2 Fig Electron Microscopy scoring data. Damage to *Salmonella* Typhimurium (STM) and *S*. Typhi (STY), expressing (Vi^+^) and not expressing Vi (Vi^-^) after exposure to human serum was visualized by electron microscopy and quantified by scoring multiple images. The total number of bacteria per frame was assessed (blue lines, right Y axis) and the number of damaged *Salmonella* counted to give the percentage of damaged cells (red lines, left Y axis). Three frames were counted. Data represent mean +/- SD.
(TIF)

S3 Fig Background levels of fluorescence following sequential incubation of *Salmonella* with PBS and secondary antibodies. The geometric mean fluorescence intensity was measured on the isogenic Vi-expressing and non-expressing *S*. Typhimurium (STM) and *S*. Typhi (STY) strains following incubation with PBS and then FITC-conjugated anti-human IgG, IgM, C3 and MAC. Values represent mean data from 3 separate experiments +/- SEM.
(TIF)

S4 Fig Anti-*Salmonella* antibody titers before and following incubation with isogenic Vi^+/-^ *S*. Typhimurium and *S*. Typhi. Levels of IgG (A) or IgM (B) against the O:4,5, O:9 and Vi *Salmonella* antigens in serum from donor 1 (S1 Table) prior to, and following, incubation with isogenic Vi-expressing or non-expressing *S*. Typhimurium (STM) or *S*. Typhi (STY) were measured by ELISA.
(TIF)

S5 Fig Levels of anti-Vi, O:9, O:4,5 and OMP (STM) antibody in the purified antibody pools. To assess the titer and presence of non-specific antibody, purified antibody preparations and fresh serum (FS) were assessed for anti-Vi, O:9, O:4,5 and *S*. Typhimurium outer membrane proteins (OMP) IgG titers by ELISA.
(TIF)

S6 Fig Correlation between purified anti-Vi and anti-O:9 antibody binding. Levels of anti-Vi and anti-O:9 binding to five wild-type *S*. Typhi isolates cultured at 9, 85 and 500mM NaCl were plotted against each other to examine the relationship between the binding of the two antibodies to the *Salmonella* surface. Correlation was calculated using Spearman’s rank.
(TIF)

S7 Fig Vi expression by isogenic Vi-expressing and -non-expressing *Salmonella* Typhi and Typhimurium strains. Vi expression levels on isogenic pairs of Vi^+/-^ *S*. Typhimurium (STM) and *S*. Typhi (STY) were measured using flow cytometry. Representative image is shown.
(TIF)

S1 Table Demographic data for human serum donors in study. Human serum donors used in this study were of mixed age, sex and race. No donors had reported or confirmed previous *Salmonella* infection. Three donors had received ViPS vaccine more than three years previous to venesection.
(TIF)
